# A hydroquinone-specific screening system for directed P450 evolution

**DOI:** 10.1007/s00253-018-9328-3

**Published:** 2018-09-06

**Authors:** Alexandra M. Weingartner, Daniel F. Sauer, Gaurao V. Dhoke, Mehdi D. Davari, Anna Joëlle Ruff, Ulrich Schwaneberg

**Affiliations:** 1 Institute of Biotechnology, RWTH Aachen University, Worringerweg 3, 52074 Aachen, Germany; 2 DWI – Leibniz Institut für Interaktive Materialien, Forckenbeckstraße 50, 52074 Aachen, Germany

**Keywords:** P450 BM3, Hydroquinone, Aromatic hydroxylation, Screening assay, Protein engineering, Directed evolution

## Abstract

The direct hydroxylation of benzene to hydroquinone (HQ) under mild reaction conditions is a challenging task for chemical catalysts. Cytochrome P450 (CYP) monooxygenases are known to catalyze the oxidation of a variety of aromatic compounds with atmospheric dioxygen. Protein engineering campaigns led to the identification of novel P450 variants, which yielded improvements in respect to activity, specificity, and stability. An effective screening strategy is crucial for the identification of improved enzymes with desired characteristics in large mutant libraries. Here, we report a first screening system designed for screening of P450 variants capable to produce hydroquinones. The hydroquinone quantification assay is based on the interaction of 4-nitrophenylacetonitrile (NpCN) with hydroquinones under alkaline conditions. In the 96-well plate format, a low detection limit (5 μM) and a broad linear detection range (5 to 250 μM) were obtained. The NpCN assay can be used for the quantification of dihydroxylated aromatic compounds such as hydroquinones, catechols, and benzoquinones. We chose the hydroxylation of pseudocumene by P450 BM3 as a target reaction and screened for improved trimethylhydroquinone (TMHQ) formation. The new P450 BM3 variant AW2 (R47Q, Y51F, I401M, A330P) was identified by screening a saturation mutagenesis library of amino acid position A330 with the NpCN assay. In summary, a 70-fold improved TMHQ formation was achieved with P450 BM3 AW2 when compared to the wild type (WT) and a 1.8-fold improved TMHQ formation compared to the recently reported P450 BM3 M3 (R47S, Y51W, A330F, I401M).

**Electronic supplementary material:**

The online version of this article (10.1007/s00253-018-9328-3) contains supplementary material, which is available to authorized users.

## Introduction

Dihydroxylated benzenes as hydroquinones (HQs) and catechols are versatile intermediates in organic chemistry with a variety of applications. HQs are used as developing agents in photography, as stabilizers (e.g., fuels and paints), antioxidants, bleaching agents, and chemical building blocks (Enguita and Leitão 2013; Hudnall 2000). About 50,000 tons of HQ are produced annually with increasing demand worldwide (Kannan 2006). The three most common processes for the production of HQ are hydroperoxidation of 1,4-diisopropylbenzene, hydroxylation of phenol, and oxidation of aniline (Hudnall 2000). The Hock oxidation of 1,4-diisopropylbenzene represents the most frequently used synthesis route (Kannan 2006; Rappoport 2003). Within the last decades, improvements were undertaken to reduce the number of required synthetic steps and minimize salt streams (Ran et al. 2001). Nevertheless, all chemical production routes raise environmental and safety concerns as strong acids (perchloric, trifluoromethanesulfonic, or sulfuric acids), sensitive explosive compounds (acetone peroxides), metal catalysts (e.g., Pd, TiO_2_), or hydrogen peroxide (H_2_O_2_) are needed (Costantini et al. 1996; Krumenacker et al. 2000). Miscellaneous HQ production routes have been reported, e.g., the direct electrochemical hydroxylation of phenols (Rautenbach 2002) or the oxidation of benzenes by iron N-heterocyclic carbene complexes and H_2_O_2_ (Lindhorst et al. 2017). Alternative synthesis routes are demanded; however, the direct and selective hydroxylation of aromatic compounds is one of the most challenging reactions in synthetic chemistry. Thus, the application of biocatalysts opens up on new synthesis routes with high expectations (Ullrich and Hofrichter 2007). Several biocatalytic examples for aromatic oxidation with molecular oxygen at ambient air and under mild conditions (e.g., in water; ambient temperature) are reported. Yoshida et al. (1990) reports the direct production of HQ from phenol with a *n*-butane-assimilating *Mycobacterium* (Yoshida et al. 1990). The aromatic peroxygenase (APO) from *Agrocybe aegerita* is known to catalyze benzene hydroxylation (Karich et al. 2013). Furthermore, quinic acid was produced from glucose in *Escherichia coli* which was subsequent oxidized to HQ (Ran et al. 2001). Recently, the heme-dependent monooxygenase P450 BM3 has gained great interest regarding the hydroxylation of diverse benzenes (Dennig et al. 2013; Munday et al. 2017) and accepts a huge variety of substrates (Bernhardt 2006; Urlacher and Girhard 2012; Whitehouse et al. 2012). P450 BM3 was reported to produce HQs from benzenes and phenols (Dennig et al. 2017; Sulistyaningdyah et al. 2005). Over the past decade, P450 BM3 has been successfully used in several protein engineering campaigns in research groups all over the world (Arnold 2009; Dennig et al. 2012; Li et al. 2001). P450 BM3 has been extensively engineered by directed evolution and rational design (McIntosh et al. 2014; Whitehouse et al. 2012). Protein engineering studies disclosed novel P450 BM3 variants with new substrate scope, altered regio- and stereoselectivity, and resistance in organic cosolvents (O’Reilly et al. 2011). One major hurdle in protein engineering campaigns is the detection of improved variants within larges mutant libraries (Arnold and Georgiou 2003). Valid screening assays with high sensitivity, low standard deviation, efficient throughput, and easy handling are required. Various colorimetric and fluorometric screening assays have been developed to evolve P450 BM3 for specific hydroxylation of aromatics, fatty acids, and steroids (Alcalde et al. 2004; Kille et al. 2011; Schwaneberg et al. 1999a). A frequently used screening assay in this context is the NADPH deletion assay (Glieder and Meinhold 2003). The consumption of the electron-donating cofactor NADPH is monitored during P450 catalysis through absorbance measurement at 340 nm. Nevertheless, screening of large libraries for increased NADPH oxidation generates often P450 variants with reduced coupling efficiencies and reduced product formation despite of increased NADPH consumptions (Morlock et al. 2018). Therefore, either an additional analysis of product formation (e.g., GC-FID/HPLC) or a product-specific screening assay is necessary to identify more productive variants. Apart from TLC plotter (Belsare et al. 2014), general applicable product quantification methods (e.g., GC-MS or HPLC) have not been reported for P450s to the best of our knowledge. A specific screening system for the quantification of phenol is the 4-aminoantipyrine (4-AAP) assay (Wong et al. 2005). This assay is applied in the screening of P450 BM3 libraries for variants with improved phenol production. A product-based screening system has not been reported for HQ quantification in 96-well microtiter plates (MTPs). The detection of HQs in body lotions and natural samples was described by using monomethyl-*p*-aminophenol (Murty and Murty 1981), cyanide (Ganjeloo et al. 1980), methacrylic acid (Belcher and Stephen 1951), rhodamine and ammonia (Anger and Ofri 1963), or ammonium meta-vanadate and spectroscopic methods (Uddin et al. 2011). However, all these methods are not sufficiently sensitive or compatible with biological samples to become suitable for the application in 96-well MTP screening procedures. 4-nitrophenylacetonitrile (NpCN) has been described as a reagent that forms a colored compound in the presence of traces of dihydroxylated aromatics (Légrádi 1970). NpCN is a well-studied radical inhibitor and intermediate material for synthesis of organic chemicals as dyes (Asiri 2004; Binev et al. 2000)*.* Here, we report a first screening system based on NpCN for the identification of enzyme variants with increased HQ production. Especially, our interest was focused on the detection of 2,3,5-trimethylhydroquinone (TMHQ) in P450 BM3 catalyzed reactions. TMHQ is an important building block for the synthesis of tocopherols (Lindhorst et al. 2017; Netscher 2007). We reported recently a first production route of TMHQ by P450 BM3 catalyzed dihydroxylation of pseudocumene (Dennig et al. 2017). Our aim was to screen for novel P450 BM3 variants with improved TMHQ formation and thereby validate the NpCN screening system for directed P450 BM3 evolution. Validation was achieved by screening semi-rational P450 BM3 libraries for enhanced TMHQ formation. Additionally, we demonstrated that the NpCN assay can be expanded for the detection of further dihydroxylated benzenes.

## Materials and methods

All chemicals were purchased from Sigma-Aldrich (Hamburg, Germany), Carl Roth (Karlsruhe, Germany), or Merck (Darmstadt, Germany) if not stated otherwise. Glucose dehydrogenase (GDH) from *Pseudomonas* sp. and catalase from bovine liver were obtained from Carl Roth. Salt-free oligonucleotides were obtained at HPLC purity from Eurofins MWG Operon (Ebersberg, Germany). *Dpn*I and dNTPs were purchased from New England Biolabs (Frankfurt, Germany). PhuS polymerase was produced in-house.

### Absorption spectra and linear detection range

The maximal absorption of NpCN in complex with different HQs was determined. Final concentrations of 50 μM in a total volume of 1 mL potassium phosphate buffer (KP_i_, 50 mM, pH 7.5) were used. Color formation was obtained by adding first 50 μL NpCN (0.04%, *w*/*v* in ethanol) and then 50 μL NaOH (2%, *w*/*v* in dH_2_O). Spectra were recorded from 300 to 700 nm with a Cary 50 Bio UV/vis spectrophotometer (Varian; Agilent Technologies, Darmstadt, Germany). The linear detection ranges of several HQs under screening conditions in MTPs were defined. Final HQ concentrations ranging from 0 to 1 mM (500 mM stock solution; solubilized in DMSO) in a total of 300 μL KP_i_ (50 mM, pH 7.5) were tested. Color formation was obtained by addition of 20 μL NpCN (0.04%) and 20 μL NaOH (2%). Reactions were mixed by pipetting for 10 s, and the absorbance was measured in a Tecan Sunrise MTP reader (Tecan Group AG, Männedorf, Switzerland).

### Saturation mutagenesis at position A330 of P450 BM3

P450 BM3 (GenBank accession number of P450 BM3 WT: WP_034650526.1) variants AW1 (vector pALXtreme-1a; R47Q, Y51F, I401M) and M2 (R47S, Y51W, I401M) (Dennig et al. 2012) were used as templates for the site saturation mutagenesis (SSM) library on position A330. Oligonucleotides were designed with following sequences: 5′- TGGCCAACTGCTCCTNNKTTTTCCCTATATGC-3′ (forward primer) and 5′-CATATAGGGAAAAMNNAGGAGCAGTTGGCCATAAGC-3′ (reverse primer). Amplification of DNA was performed using the described two-step PCR protocol (Dennig et al. 2012). The annealing temperature was adjusted to 60 °C (for 30 s). Subsequently, template DNA in the PCR was digested overnight with 20 U *Dpn*I and PCR products were purified (NucleoSpin® Gel and PCR Clean-up kit, Macherey-Nagel, Düren, Germany). The library was inserted into chemically competent *E. coli* BL21 lacI^Q1^ cells (Blanusa et al. 2010) and plated on LB agar plates supplemented with 50 μg mL^−1^ kanamycin. Plasmid purification was performed (NucleoSpinS Plasmid kit, Macherey-Nagel), and sequencing of the P450 BM3 genes was conducted at GATC Biotec (Konstanz, Germany).

### Cultivation of P450 BM3 in 96-deep-well plates

Single colonies of the P450 BM3 library were transferred into 96-well flat bottom MTPs (Greiner Bio-One GmbH, Frickenhausen, Germany) filled with LB medium (100 μL; 50 μg mL^−1^ kanamycin). Six wells of each MTP were inoculated with replicates of a negative control (empty vector) and the starting variant (P450 BM3 M2 or AW1). Cultivation was performed in a MTP shaker (Multitron II; Infors GmbH, Einsbach, Germany) for 16 h (37 °C, 900 rpm, 70% humidity). The overnight cultures were used as precultures for expression and stored at − 80 °C after addition of 100 μL sterile glycerol (50%, *v*/*v*). Library expression occurred in round bottom 2.2 mL 96-deep-well plates (Brand GmbH, Wertheim, Germany) in a volume of 600 μL terrific broth (TB) medium. The protocol and expression conditions described by Nazor et al. (2008) were followed. Cells were incubated in a MTP shaker for 20 h (30 °C, 900 rpm, 70% humidity). Expression cultures were harvested by centrifugation (15 min, 3220×*g*, 4 °C), supernatant discarded, and cell pellets stored at − 20 °C until further use.

### Screening with the NpCN assay for improved P450 BM3 variants

Frozen cells were thawed at room temperature for 10 min. Cells were disrupted by resuspending in a total of 300 μL KP_i_ (50 mM, pH 7.5) supplemented with lysozyme (2.5 mg mL^−1^). After incubation for 1 h (37 °C, 900 rpm, 70% humidity), lysed cells were centrifuged (15 min, 3220×*g*, 4 °C). Two 96-well MTPs per library (180 clones) were screened for increased NADPH oxidation rate and improved TMHQ formation each. NADPH depletion assay was performed as described by Glieder and Meinhold (2003). Reaction contained per well 20 μL cell lysate with expressed P450 BM3, 10 mM pseudocumene, 2% (*v*/*v*) DMSO, and KP_i_ in a total volume of 200 μL. MTPs were incubated for 5 min before supplementation with 50 μL NADPH (final concentration 200 μM). Oxidation of NADPH was measured at 340 nm in a Tecan Sunrise MTP reader (Tecan Group AG). After NADPH depletion, 50 μL of a NADPH regeneration mix was added (100 μM NADPH, 2 U mL^−1^ glucose dehydrogenase (GDH), and 40 mM glucose in KP_i_, all final concentrations). Plates were incubated for 2 h at 600 rpm (room temperature). For product detection, 20 μL 0.04% (*w*/*v* in ethanol) NpCN and 20 μL 2% (*w*/*v*) NaOH were added and mixed by pipetting for 10s. Absorption was measured at respective wavelength with a Tecan Sunrise MTP reader. The standard deviation of the NpCN assay was determined using 93 replicates of P450 BM3 variant M3 (Dennig et al. 2017). For the calculation of the true standard deviation, absorption values obtained for cell lysates without P450 BM3 (negative control, background) were subtracted.

### Expression and purification of P450 BM3 variants

Expression of P450 BM3 in shake flasks and purification of the monooxygenase was performed as described elsewhere (Nazor et al. 2008). Shortly, for the purification frozen cell pellets from a 250 mL culture were resuspended in 15 mL Tris/HCl buffer (100 mM, pH 7.8). Cells were homogenized by sonication for 4 min (with 30 s interval, 40% amplitude, Vibra-Cell VCX-130; Sonics, Newtown, CT, USA) and subsequently disrupted in an Avestin EmulsiFlex-C3 high-pressure homogenizer (Ottawa, ON, Canada) by applying three cycles of 1500 bar pressure. After centrifugation (30 min, 16,000×*g*, 4 °C), the supernatant was filtered with a 0.22-μm filter membrane. Purification of the P450 BM3 variants was performed by anion exchange chromatography with a Toyopearl DEAE 650S matrix (Tosoh Bioscience, Griesheim, Germany) and an ÄKTAprime chromatography system (GE Healthcare, Solingen, Germany) (Schwaneberg et al. 1999b). The purified P450 BM3 enzyme was concentrated with an Amicon centrifugation tube (30 kDa cut-off; Merck Millipore, Darmstadt, Germany) and desalted using a PD-10 desalting column (GE Healthcare) equilibrated with KP_i_ (50 mM, pH 7.5). For long-time storage, enzyme samples were shock-frozen in liquid N_2_ and lyophilized (Alpha 1–2 LD plus freeze-dryer Christ, Osterode am Harz, Germany). Twenty-four-hour conversions were performed with cell-free lysates. Therefore, frozen cell pellets were resuspended in KP_i_ (10% culture volume) and lysed by sonication for 5 min (with 30 s interval, 40% amplitude, Vibra-Cell VCX-130). Cell debris was removed by centrifugation (30 min, 12,000×*g*, 4 °C).

### Substrate conversion and kinetic characterization of P450 BM3 variants

P450 BM3 concentrations were determined by CO-binding assay following the protocol by Omura and Sato (Omura and Sato 1964). Regioselectivity, product yields, and total turnover number (TTN) were determined in presence of GDH for efficient regeneration of the NADPH cofactor. The TTN was calculated based on the total product formation after 24 h. Conversions (1 mL) contained: 1 μM P450 BM3 variant, 3 U GDH, 60 mM glucose, 1400 U mL^−1^ catalase, 10 mM substrate, 2% (*v*/*v*) DMSO, 400 μM NADPH, 10 mM ascorbic acid, and KP_i_ (50 mM, pH 7.5). Ascorbic acids were added to prevent oxidation of TMHQ to the respective 1,4-benzoquinone. Kinetic characterizations were performed with purified P450 BM3. The reactions contained 10 mM pseudocumene and 2% (*v*/*v*) DMSO in a final volume of 1 mL KP_i_. After 5 min incubation, NADPH was supplemented and the oxidation of the cofactor was measured at 340 nm in a spectrophotometer (Varian Cary 50 UV). NADPH oxidation rates were determined using 200 μM NADPH and 0.1–1 μM P450 BM3 (1 μM WT, 0.5 μM AW1, 0.2 μM AW2, and 0.1 μM M3). For the determination of coupling efficiencies, 1 mM NADPH was used. The conversions were stopped with 100 μL 37% HCl after respective reaction times (2 h or 24 h) or after depletion of NADPH. Products were extracted with 500 μL *tert*-butyl methyl ether containing 2% (*w*/*v*) 2,5-dimethylphenol as internal standard. Organic phases were dried over anhydrous MgSO_4_ and analyzed by GC-FID (gas chromatography with flame-ionization detector) (Shimadzu GmbH, Duisburg, Germany). Calibration curves for all products were prepared with analytical standards. Products resulting from P450 BM3 conversions were separated using the following program: 120 °C for 8 min, heating 7 °C min^−1^ up to 210 °C, and hold for 2 min at 210 °C (Hydrodex-ß-TBDAc column, Macherey-Nagel).

### Molecular modeling

The starting structure of P450 BM3 WT was taken from the crystal structure of cytochrome P450 BM3 with the heme domain (PDB ID: 1BU7) (Sevrioukova et al. 1999). The swap function in Yasara Structure version 17.4.17 (Krieger and Vriend 2014) was used to construct catalytically competent P450 BM3 AW2 and M3. In order to minimize the substituted residue, rotamer library search was carried out using SCWRL (Wang et al. 2008). The protein residues were treated using the AMBER ff99 (Wang et al. 2000). The ligand atoms were treated using GAFF (Duan et al. 2003; Wang et al. 2004) with AM1-BCC partial charges (Jakalian et al. 2002) employing particle mesh Ewald (Essmann et al. 1995) for long-range electrostatic interactions and a direct force cutoff of 10.5 Å. For molecular docking, crystal water molecules were deleted except the one which is coordinating to the iron of the heme domain. The constructed models were minimized using a water box, first with steepest descent and then simulated annealing (time step of 2 fs, atom velocities scaled down by 0.9 every 10th step) starting from 98 K, 198 K and 298 K with a time averaged Berendsen thermostat until convergence was reached. The minimized models were further used for molecular docking studies. A grid box of 12 Å around the active site was applied by centering heme iron of P450 BM3. Molecular docking calculations were performed using Autodock4.2 plug-in within Yasara with a fixed protein backbone. The side chains of the neighboring residues which are within 5 Å from the bound water molecule were treated flexible. One hundred docking runs were carried out and the docking solutions were clustered applying a RMSD cutoff of 0.5 Å and using the default settings provided within the YASARA dock_run macro file.

## Results

Here, we report for the first time a 96-well MTP screening system for the detection of dihydroxylated aromatics obtained in P450-catalyzed reactions. In a first step, the interaction of NpCN with different HQ is reported. Then, optimized parameters of the NpCN assay for application as a versatile MTP-based screening system are given. Finally, the applicability of the NpCN screening system was validated in a semi-rational protein engineering campaign by screening P450 BM3 libraries for identification of variants with improved TMHQ formation.

### Proposed reaction of NpCN with HQ

**Fig. 1:**
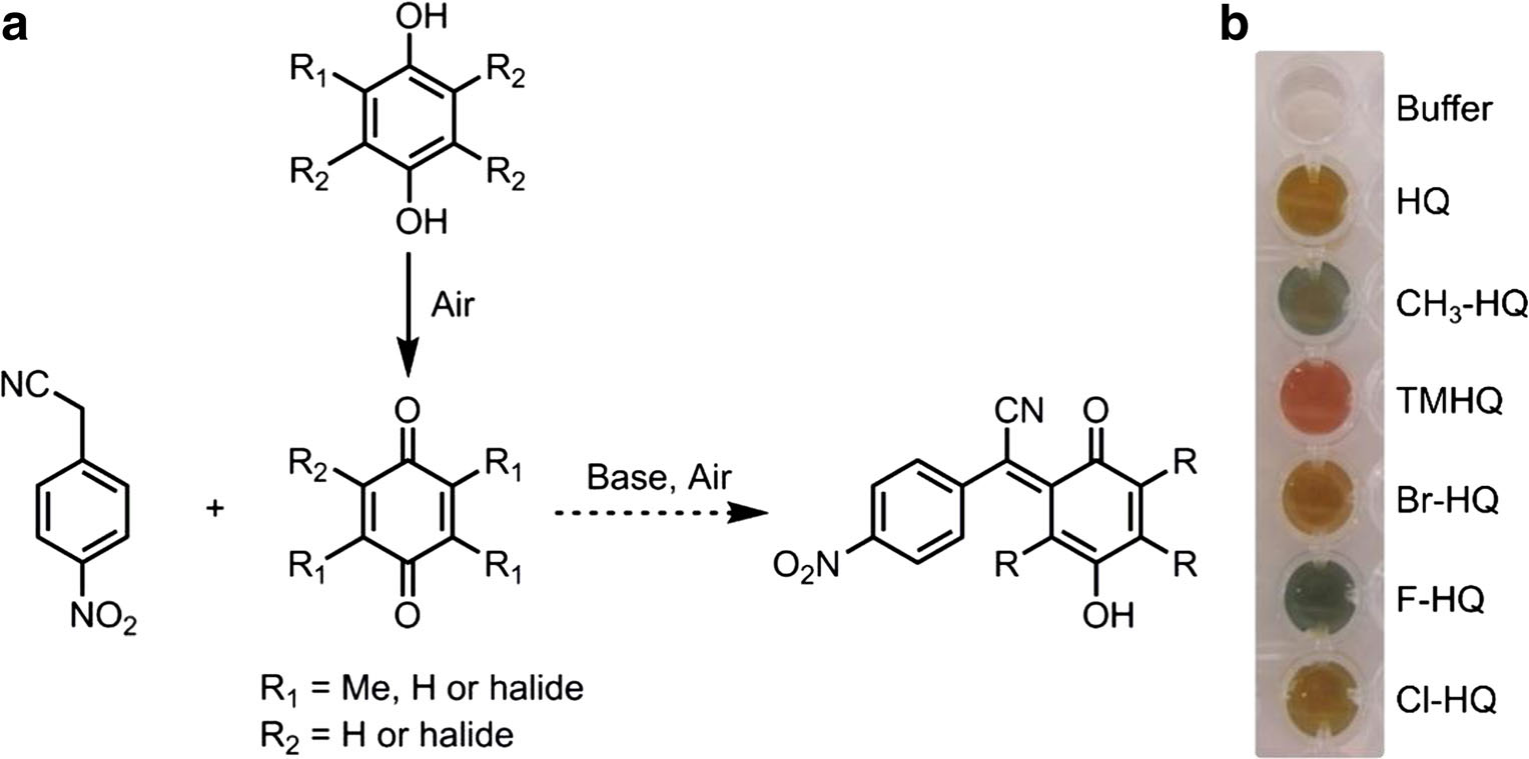
Plausible product formation of NpCN with HQ (**a**). Under basic conditions and under air a colorimetric detectable complex is formed. Color formation is depended on different HQs (**b**): CH_3_-HQ methylhydroquinone, TMHQ trimethylhydroquinone, Br-HQ bromohydroquinone, F-HQ fluorohydroquinone, Cl-HQ chlorohydroquinone. HQ concentrations were 50 μM in a total of 300 μL phosphate buffer. Color formation was obtained directly after adding 20 μL (0.04%) NpCN and 20 μL (2%) NaOH

The NpCN assay allows the detection of a wide range of dihydroxylated aromatics. NpCN is a sensitive reagent for the detection of quinones, HQ, and pyrocatechols (Légrádi 1970). Figure 1a shows the proposed dye formed when NpCN interacts with HQ under alkaline conditions under air. Proton NMR analysis and EI-MS of the extracted reaction mixture support the proposed product formation (Figs. S1 and S2). Performing the reaction under air-exclusion led to isolation of the starting materials.

We obtained a color formation for HQs, catechols and benzoquinones (Table 1). Interaction of NpCN with HQs in presence of NaOH led to a blue, green, brown, or red color development (Fig. 1b). In contrast, no color formation was visible for tetramethylhydroquinone, phenols, resorcinol, benzyl alcohols, and cyclohexanediol (Table 1). We account this finding to the ability of HQs and catechols to undergo oxidation with air to the corresponding benzoquinones that offer the Michael acceptor position necessary for the reaction with NpCN (Fig. 1).

**Table 1:** Aromatic compounds and their interaction with NpCN

Color formation	No color formation
Bromohydroquinone	Benzenes
Chlorohydroquinone	1,4-Cyclohexonediol
2-Chloro-1,4-benzoquinone	1,3-Dihydroxynaphtalene
2,5-Dimethyl-1,4-benzoquinone	Dimethylbenzylalcohols
Fluorocatechol	Hydroxymethyl phenols
Fluorohydroquinone	Phenols
Hydroquinone	Resorcinol
Methylhydroquinone	Tetramethylhydroquinone
2-Methyloxyhydroquinone	
Pyrocatechol	
Tetrachloro-1,4-benzoquinone	
TMHQ	

A color formation of aromatic compounds (1 mM) on the left side was obtained in presence of NpCN under basic conditions. No color formation was visible for the compounds on the right side. Compounds are sorted in alphabetical order. For chemical structures, see Table S4

The maximal absorption wavelength of the complex is specific for each HQ and depends on the substitutions on the aromatic ring. The absorption maxima we obtained in the presence of NpCN were 520 nm for TMHQ, 420 nm for HQ, 430 nm for Br-HQ, and 450 nm for CH_3_-HQ, respectively (50 μM HQ in 1 mL potassium phosphate buffer; Fig. S3).

### NpCN assay for screening of enzyme libraries

**Fig. 2:**
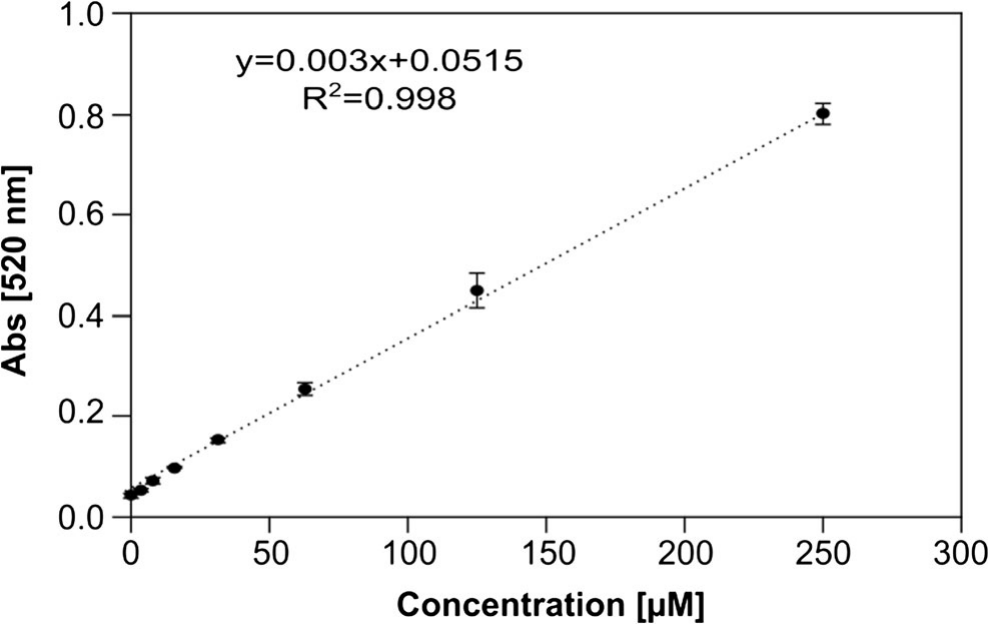
Linear detection range of the NpCN assay in a 96-well MTP format: at 520 nm, a linear response is achieved between 5 and 250 μM TMHQ

The hydroxylation of pseudocumene by P450 BM3 was selected as target reaction for the establishment of the NpCN assay. The hydroxylation of pseudocumene by P450 BM3 leads to the formation of six monohydroxylated products as well as TMHQ (Fig. S4) (Dennig et al. 2017). TMHQ is a key tocopherol precursor and of commercial interest (Netscher 2007). We aimed to identify novel P450 BM3 variants with improved TMHQ formation. Therefore, a screening assay that is specific for HQs but shows no color formation in presence of monohydroxylated benzenes is a prerequisite. NpCN as reactant led to color formation with TMHQ but not with trimethylphenols (TMP) or dimethylbenzylalcohols (Table 1; Fig. S5). Originally, the interaction of NpCN with HQ was described to occur in ethanol (Légrádi 1970). In contrast, P450s require aqueous systems for catalysis of hydroxylation reactions (Reinen et al. 2015; Whitehouse et al. 2012). The assay conditions were, thus, adjusted for the application in phosphate buffer (50 mM, pH 7.5) and downscaled into MTP format. Different volumes and concentrations of both, NpCN and NaOH, were investigated (0.1–10% NaOH, 0.006–0.2% NpCN, data not shown). Most appropriate results were obtained with 20 μL 0.04% (*w*/*v*) NpCN and 20 μL 2% (*w*/*v*) NaOH added to a reaction volume of 300 μL (phosphate buffer). Under these conditions, TMHQ concentrations showed a linear response from 5 to 250 μM at 520 nm (Fig. 2). A linear detection range of other HQs (HQ, Br-HQ, CH_3_-HQ, and Cl-HQ) in the presence of NpCN was determined to span from 1 μM to 100 μM (Fig. S6).

**Fig. 3:**
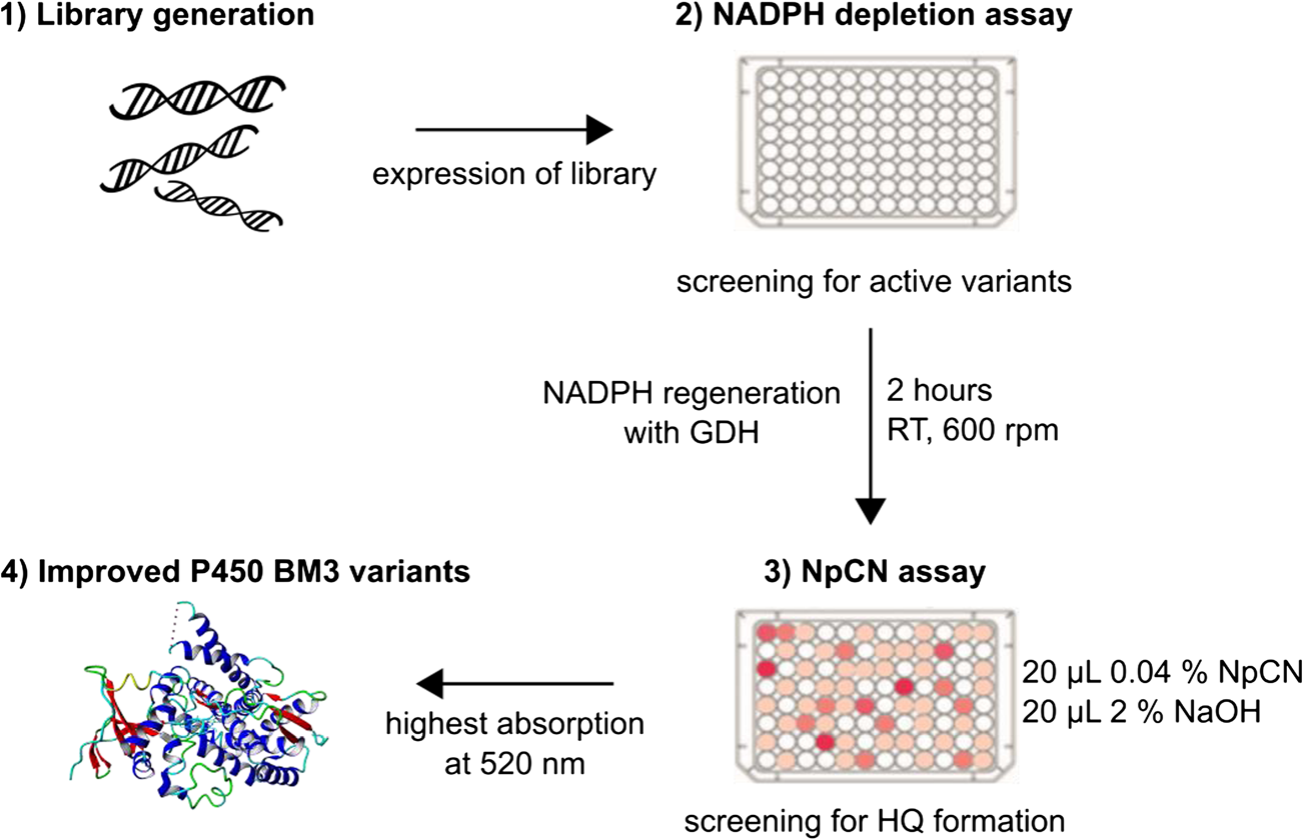
Strategy for P450 evolution by applying the NpCN assay in combination with the NADPH depletion assay. Enzyme libraries (1) are expressed in a suitable host (*E. coli*) and screened with the widely used NADPH depletion assay (2) for active P450 variants. GDH is supplemented to regenerate NADPH and enable product formation for a longer time frame. The NpCN assay (3) is performed by supplementing NpCN and NaOH. Absorption is measured at the wavelength specific for the respective HQ (520 nm for TMHQ). P450 BM3 variants showing the highest absorption values (4) are further characterized

**Fig. 4:**
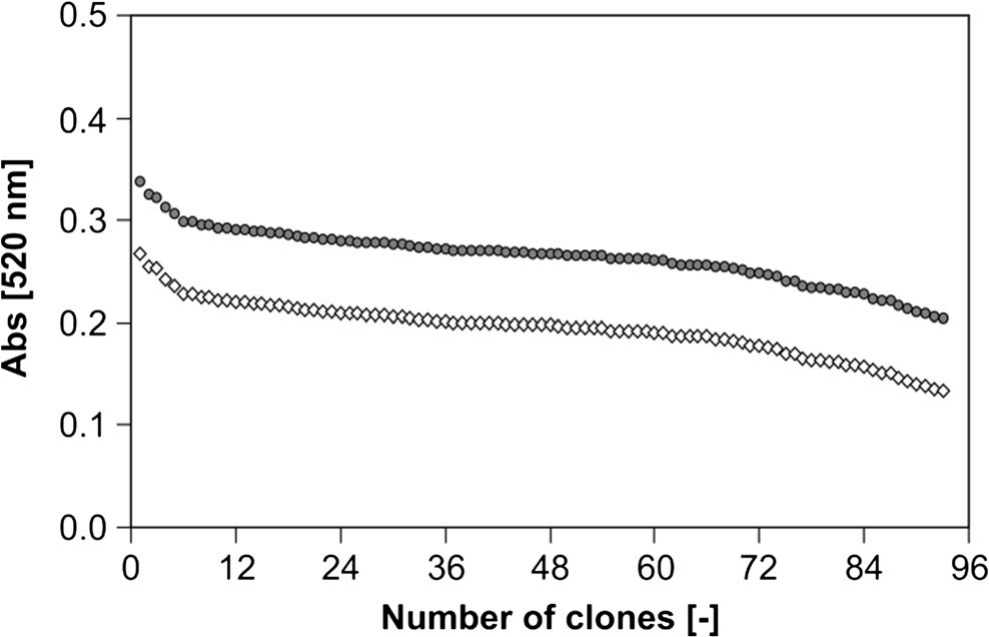
Standard deviation of the NpCN assay. Measured absorption values at 520 nm in descending order of P450 BM3 variant M3 catalyzed conversion of pseudocumene in a 96-well plate. In dark gray, the apparent standard deviation (10%) is depicted. The white diamonds show the true standard deviation (14%) after subtraction of the empty vector background

We applied the NADPH depletion assay (Glieder and Meinhold 2003) in combination with the NpCN assay. The used NADPH concentration (200 μM) was not sufficient to detected TMHQ formation starting from pseudocumene with the available P450 BM3 variants. Thus, GDH (2 U mL^−1^) and glucose were supplemented for NADPH regeneration to achieve longer conversion times (Fig. 3). We investigated different NADPH regeneration times (0.5–4 h) and 2 h turned out to be suitable for screening toward TMHQ formation. The standard deviation of the NpCN assay after 2 h was 10% using the P450 BM3 M3 (R47S, Y51W, A330F, I401M). After subtraction of the background (lysate without P450 BM3), a true standard deviation of 14% was obtained (Fig. 4). Standard deviations below 15% are routinely employed in successful directed evolution campaigns (Cheng et al. 2015; Wong et al. 2005).

Furthermore, the application of the NpCN assay is not restricted to P450s and can be adjusted for the screening of other enzyme classes, e.g., peroxygenases as well. In preliminary experiments the conversion of phenol and 3-chlorophenol by the unspecific peroxygenase (UPO) from *Agrocybe aegerita* (UPO variant PaDa-I; Molina-Espeja et al. 2014) led to color formation when applying the NpCN assay (Fig. S7).

### Evolution of P450 BM3 for increased TMHQ formation

**Fig. 5:**
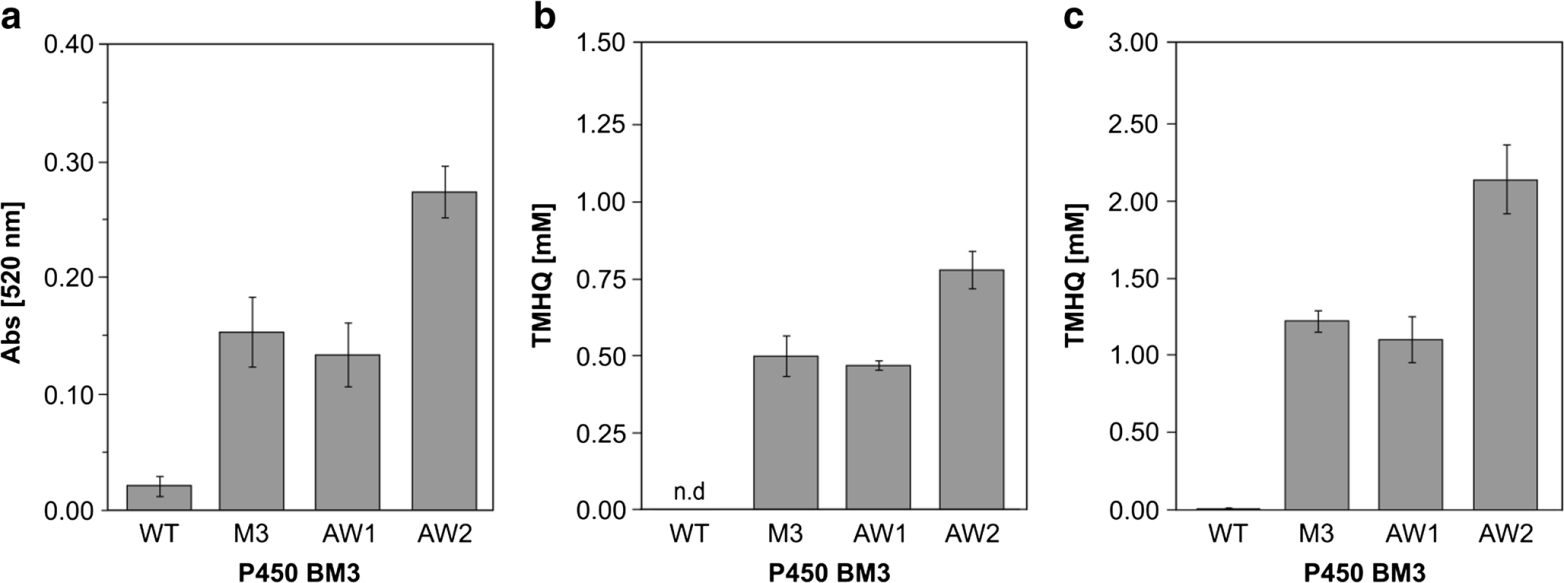
TMHQ formation of different P450 BM3 variants detected with the NpCN assay and GC-FID. **a** NpCN assay; absorption at 520 nm obtained for different P450 BM3 variants (pseudocumene as a substrate, 2 h NADPH regeneration with GDH). **b** TMHQ detected by GC-FID after 2 h pseudocumene conversion, n.d. not detected. **c** TMHQ concentrations obtained by GC-FID after 24 h pseudocumene conversion

Two SSM libraries of P450 BM3 were screened for improved TMHQ formation to validate the NpCN assay. As a first starting variant, we used P450 BM3 AW1 (R47Q, Y51F, I401M) and saturated position 330 to all 20 canonical amino acids. Position 330 was reported to have an influence on the activity of P450 BM3 for aromatic hydroxylations (Dennig et al. 2017; Munday et al. 2017). We screened in total 180 variants (theoretical calculated > 95% diversity coverage) (Firth and Patrick 2008) with the NpCN assay (Fig. S8) resulting in a preferred substitution from alanine to proline at position 330. The obtained P450 BM3 AW2 (R47Q, Y51F, I401M, A330P) was subsequently characterized in detail. We performed pseudocumene conversions in 1 mL volume in presence of a cofactor regeneration system (GDH) and investigated the product formation with GC-FID (Fig. S9). After 2 h and 24 h, P450 BM3 AW2 showed a 1.5- to 2-fold improved TMHQ formation compared to P450 BM3 AW1 (2.2 mM vs 1.1 mM TMHQ for 24 h, Table S1). The absorption values obtained with the NpCN assay correlated well with the GC-FID results (Fig. 5). For further validation, we compared P450 BM3 WT and the recently described P450 BM3 M3 (Dennig et al. 2017) with P450 BM3 AW2. Increased absorption values (absorbance of 0.29 vs 0.15 at 520 nm) were detected for P450 BM3 AW2 compared to M3 (Fig. 5a; NpCN assay). Analysis of the TMHQ formation by GC-FID confirmed the improved TMHQ production of the P450 BM3 AW2 (Fig. 5b, c). Under constant NADPH regeneration, P450 BM3 AW2 produced up to 2.2 mM TMHQ whereas the P450 BM3 M3 produced 1.2 mM TMHQ (Fig. 5; Table S1). These results prove that it is possible to identify P450 BM3 variants due to their capability to produce HQs with the developed NpCN assay in MTP format.

Additionally, we screened an SSM library on position A330 starting from P450 BM3 M2 (R47S, Y51W, I401M) (Dennig et al. 2012, 2017) with the NpCN assay (Fig. S10). P450 BM3 M2 was in previous studies engineered for aromatic hydroxylation of *p*-xylene (Dennig et al. 2012). In our last report (Dennig et al. 2017), the screening of the SSM library on position A330 was performed with the NADPH depletion assay and the 4-AAP assay (specific for phenols) (Wong et al. 2005). Screening led to the identification of P450 BM3 M3 that showed the same ability to produce TMHQ (0.18 g L^−1^) as the P450 BM3 M2 but had a significant higher NADPH oxidation rate and higher coupling efficiency (Dennig et al. 2017). Screening for improved TMHQ formation with the NpCN assay revealed the P450 BM3 AW3 (R47S, Y51W, A330P, I401M). In comparison to P450 BM3 M3, the variant AW3 with substitution A330P showed a 1.3-fold increased TMHQ formation (Fig. S11; Table S1). These results underline the importance to screen for the desired end-product and prove the applicability and superiority of a product based screening system such as the NpCN assay.

### Characterization and substrate binding of P450 BM3 variant AW2

**Fig. 6:**
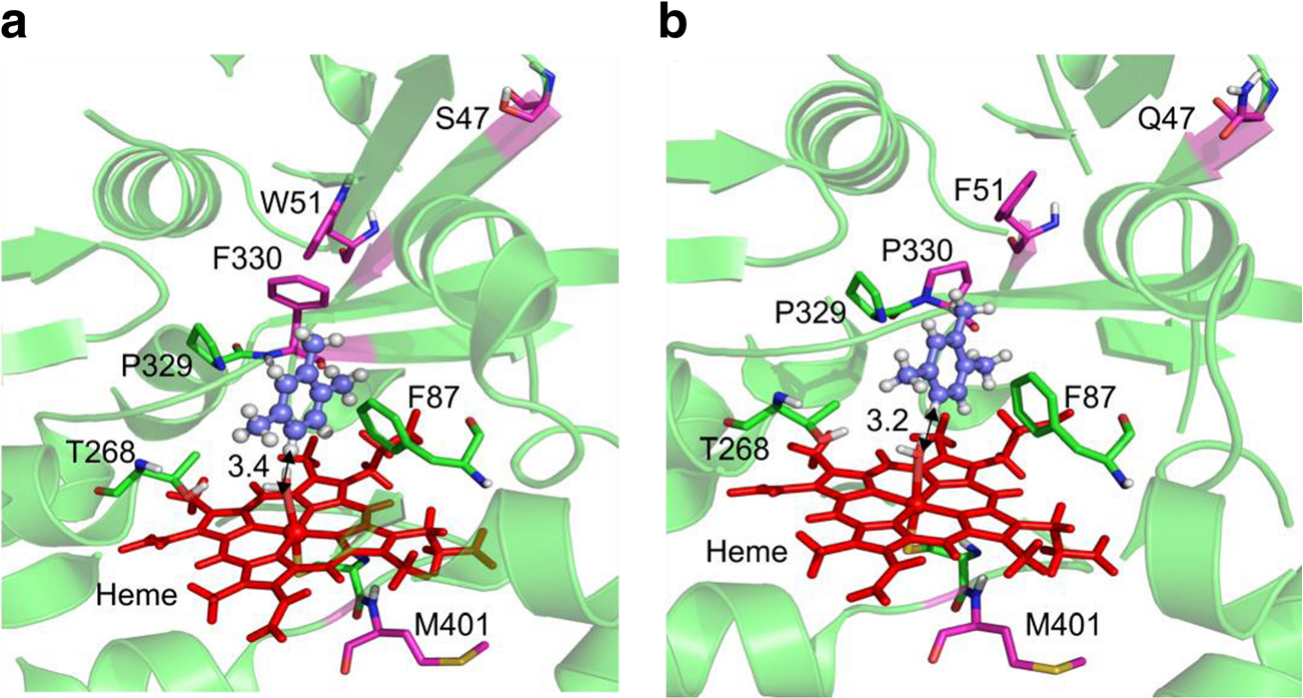
Molecular docking pose of pseudocumene in the active site of **a** P450 BM3 M3 (R47S, Y51W, A330F, I401M; binding energy − 10.28 kcal mol^−1^) and **b** P450 BM3 AW2 variant (R47Q, Y51F, A330P, I401M; binding energy − 11.18 kcal mol^−1^). Reciprocal arrows indicate the closest distance (in Å) between heme-bound water ligand and closest aromatic C-atoms of pseudocumene. Pseudocumene is shown as ball and stick, whereas all the active site residues including heme are shown as sticks

We determined the by-product formation and the catalytic performance of P450 BM3 WT and AW1, AW2, and M3. High uncoupling results in increased H_2_O_2_ formation which often reduces the TTNs of P450s (Jung et al. 2011). P450 BM3 AW3 did not show any improvements compared to P450 BM3 AW2 and was not investigated further (Fig. S11; Table S1). The formation of the intermediates 2,3,5-TMP and 2,3,6-TMP was improved 3.5-fold by P450 BM3 AW2 compared to M3 (M3 = 0.037 g L^−1^, AW2 = 0.13 g L^−1^). 2,3,5-TMP and 2,3,6-TMP are both desired products as both can be further hydroxylated to TMHQ (Fig. S4). Less by-product formation of 2,4,5-TMP was obtained for P450 BM3 AW2 compared to AW1 and M3 (AW2 = 13%, AW1 = 37%, and M3 = 29%) (Table S2). The coupling efficiency of P450 BM3 AW2 and M3 was similar (37% vs 40%; Table S3) and significantly improved when compared to the P450 BM3 AW1 and WT (32% for AW1 and 21% for WT). P450 BM3 M3 reached a total product concentration of 0.44 g L^−1^ corresponding to a TTN of 4330 (Table S3). The P450 BM3 AW2 showed an improved TTN of 7041 (total product formation 0.69 g L^−1^). This makes P450 BM3 AW2 a better catalyst for pseudocumene hydroxylation and TMHQ formation. Additionally, molecular docking studies were performed to understand the rationale behind the improvements of P450 BM3 AW2. It is known that close interactions of the substrate and the heme-bound water molecule are crucial to initiate the catalytic cycle in P450-catalyzed reactions (de Visser and Shaik 2003), and a strong binding of the substrate is reported to contribute to high coupling efficiency (Munro et al. 2002). For assessment of the docking results, we considered the distance between heme-bound water molecule and closest aromatic C-atoms of pseudocumene. Figure 6 shows the binding orientation of pseudocumene in the binding pockets of P450 BM3 M3 and AW2. The binding of pseudocumene to P450 BM3 AW2 (− 11.18 kcal mol^−1^) is stronger as compared to variant M3 (− 10.28 kcal mol^−1^). Similarly, the distance between heme-bound water molecule and the closest C-atom is decreased (3.2 Å) in case of P450 BM3 AW2 compared to M3 (3.4 Å). The closer and stronger binding of pseudocumene to P450 BM3 AW2 is in good agreement with the measured higher TTN. Additionally, the phenyl ring of F330 (A330F) in P450 BM3 M3 impeded the substrate access (Fig. S12), whereas in P450 BM3 AW2, the smaller proline substitution at position 330 (A330P) kept the substrate access channel open. Positions 47 and 51 located at the entrance of the substrate access channel further extended the channel when substituted with glutamine (R47Q) and phenylalanine (Y51F). Furthermore, in case of P450 BM3 AW2, the length of the substrate access channel was shorter (27.24 Å) compared to the M3 variant (28.36 Å).

## Discussion

A first screening system for the detection of different HQs in P450-catalyzed reactions was developed and validated in a semi-rational evolution campaign by screening P450 BM3 variants for increased TMHQ formation. The NpCN screening system is based on the interaction of HQ with NpCN under basic conditions (Fig. 1a). The NpCN assay can be applied for the quantification of many HQs (Table 1; Fig. 1b). Key parameters for a robust and reliable screening system were determined. In detail, the NpCN assay has a broad linear detection range and high sensitivity (5 to 250 μM), making it suitable to screen for enzymatic improvement in terms of product formations. To validate the NpCN screening system, the hydroxylation of pseudocumene by P450 BM3 was selected to screen for increased TMHQ formation. With pseudocumene as a substrate, we obtained standard deviations of 14% for the P450 BM3 M3 (Fig. 4). P450 BM3 M3 served as positive control because P450 BM3 WT produces only negligible amounts of TMHQ (Dennig et al. 2017) (Fig. 5). A standard deviation of 12% was obtained for 93 replicates of P450 BM3 AW2 (Fig. S13). As a comparison, standard deviations of the 4-AAP assay for the detection of phenols are ranging from 10.6 to 15% depending on the substrate used (Wong et al. 2004). Standard deviations are strongly influenced by P450 expression levels and cultivation conditions in deep-well plates (Arnold and Georgiou 2003) as well as by substrate insolubility (pseudocumene 0.4 μmol L^−1^; McAuliffe 1966). In general, deviations of 10 to 15% proved to be sufficient to conduct successful directed evolution campaigns (Cheng et al. 2015; Wong et al. 2005). The NpCN assay was designed to be as close as possible to desired final reaction conditions in terms of NADPH regeneration, buffer conditions, and substrate concentration. The NADPH depletion assay (Glieder and Meinhold 2003) can be used in combination with the NpCN assay (Fig. 3). The detection of formed HQs by the NpCN assay can occur directly after the NADPH depletion assay when sufficient HQ is formed and the product formation is within the linear detection window. If the concentration of dihydroxylated products is under the detection limit, GDH and glucose can be supplemented for NADPH regeneration to achieve higher conversion (Fig. 3). In case of phenols as substrate, the formation of dihydroxylated products may be detected immediately after the NADPH depletion assay, as only one hydroxylation step is required. We choose 2 h as conversion time (NADPH regeneration) to reduce the overall assay time and ensure a successful selection for improved P450 BM3 variants (Fig. 5). Depending on the enzyme and substrate applied, conversion times need to be adjusted. The screening of two SSM libraries on position A330 (starting variants P450 BM3 AW1 and M2) with the NpCN assay revealed a preferred substitution of alanine to proline. P450 BM3 AW2 had a 2-fold increased TMHQ formation (up to 2.2 mM TMHQ) compared to the starting P450 BM3 AW1 and a 1.8-fold improved TMHQ formation compared to the recently described P450 BM3 M3 (Fig. 5; Table S1). Previous study of pseudocumene with P450 BM3 M3 revealed that the introduction of phenylalanine at position 330 introduces an additional π-π interaction. This interaction improves the productivity and coupling efficiency, as well as the selectivity toward aromatic hydroxylation of pseudocumene (Dennig et al. 2017). Furthermore, it has been reported that the A330P substitution constrained the active site by repositioning of side chain of P329 into the substrate access channel resulting in increased coupling efficiency and activity of P450 BM3 toward small molecules such as toluene, propylbenzene, and 3-methylpentane (Whitehouse et al. 2009, 2010). Our docking studies are in agreement with our experimental data, and let us assume that the binding of pseudocumene in the active site of P450 BM3 AW2 (− 11.18 kcal mol^−1^) is more favorable compared to P450 BM3 M3 (− 10.28 kcal mol^−1^).

In summary, a product- and hydroquinone-specific screening system was developed and validated by screening of SSM libraries, yielding P450 BM3 AW2 with a TMHQ formation of up to 2.2 mM. The specific activity of AW2 is 70-fold higher in the production of TMHQ than P450 BM3 WT. The low detection limit (5 μM), the broad linear detection range (5 to 250 μM), and broad substrate scope (different aromatics) make the NpCN screening system broadly applicable to detect aromatic hydroxylation.

## Electronic supplementary material

ESM 1 The supporting material includes screening results, catalytic characterization of P450 BM3 variants and GC-FID, EI-MS and NMR data. (PDF 892 kb)
