# Supplementary material for: A hydroquinone-specific screening system for directed P450 evolution
Source: Appl Microbiol Biotechnol. 2018 Sep 6;102(22):9657–67. doi: 10.1007/s00253-018-9328-3 (PMC6208966; doi:10.1007/s00253-018-9328-3)
Supplement: Supplementary file 1 — The supporting material includes screening results, catalytic characterization of P450 BM3 variants and GC-FID, EI-MS and NMR data. (PDF 892 kb) [file 253_2018_9328_MOESM1_ESM.pdf]

Applied Microbiology and Biotechnology

Electronic Supplementary Material for:

A hydroquinone specific screening system for directed P450 evolution

Alexandra M. Weingartner[1], Daniel F. Sauer[1], Gaurao V. Dhoke[1], Mehdi D. Davari[1], Anna Joëlle Ruff[1] and Ulrich Schwaneberg[1,2*]*

[1] RWTH Aachen University, Institute of Biotechnology, Worringerweg 3, 52074 Aachen, Germany

[2] DWI –Leibniz Institut für Interaktive Materialien, Forckenbeckstraße 50, 52074 Aachen, Germany

*Corresponding authors:

Prof. Dr. Ulrich Schwaneberg
Tel.: +49 241 80 24170
E-Mail: u.schwaneberg@biotec.rwth-aachen.de

Dr. Anna Joëlle Ruff
Tel.: +49 241 80 23604
E-Mail: aj.ruff@biotec.rwth-aachen.de

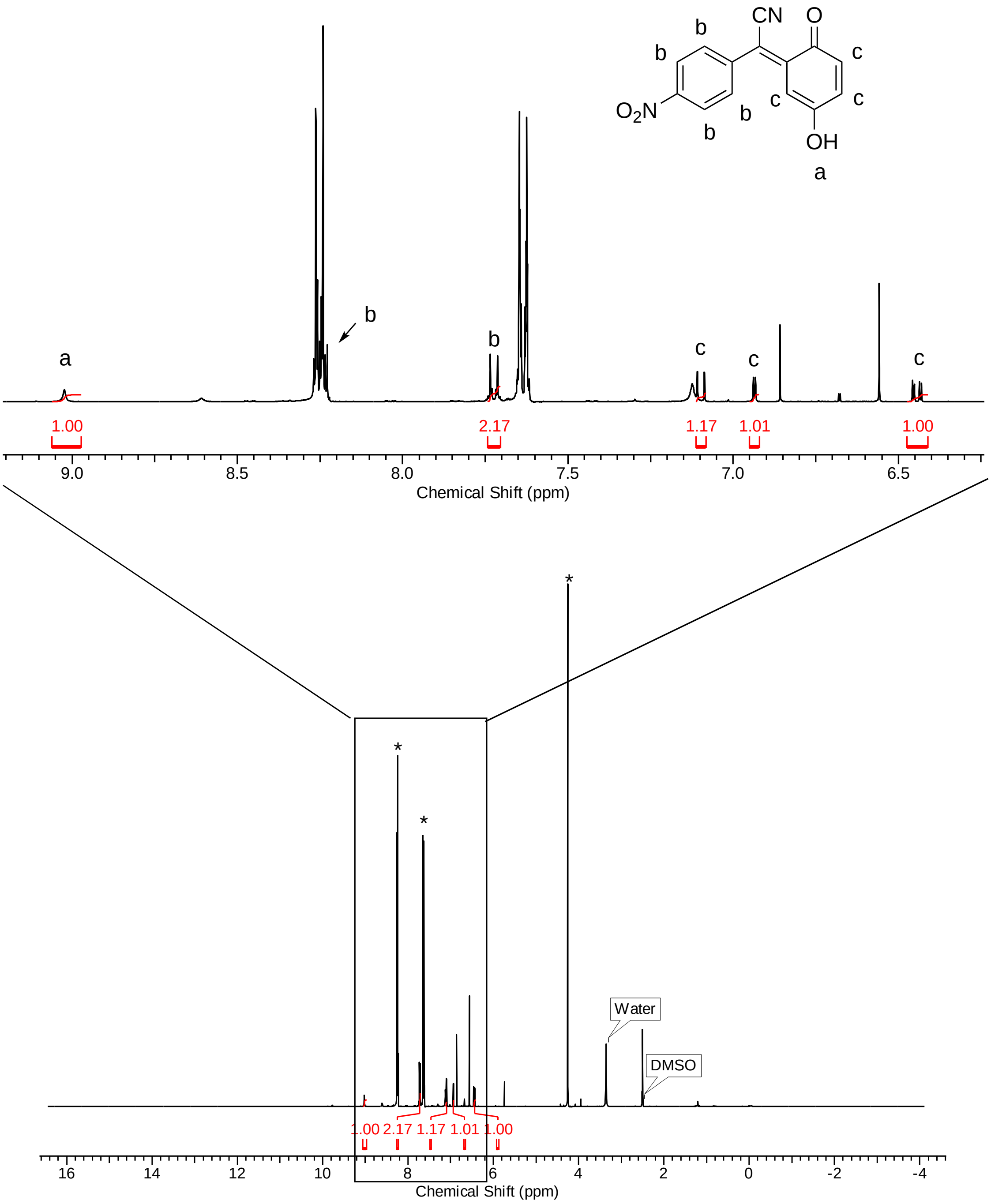

Fig. S1 ^{1}H NMR spectrum (400 MHz, DMSO-d6, 23°C) of the extracted reaction mixture of HQ and NpCN. The signals designated with an asterisk (*) indicate NpCN. The zoomed spectrum above shows the reaction product of HQ and NpCN with designated signals. The NMR spectrum was recorded on a Bruker DRX 400 spectrometer. Chemical shifts were referenced internally by using the residual solvent resonances (Fulmer et al. 2010)

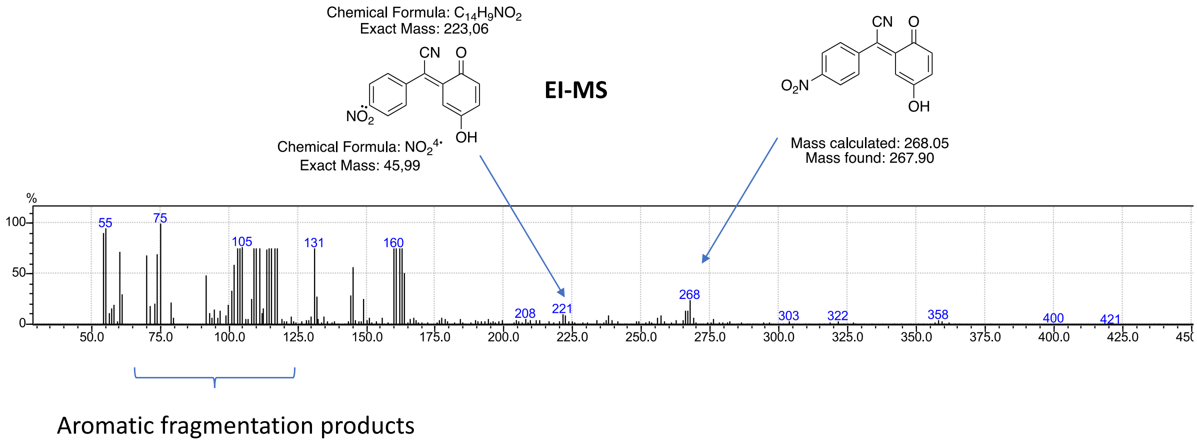

Fig. S2 EI-MS of the extracted reaction mixture of HQ and NpCN. EI-MS was recorded on a GCMS-QP2010 from Shimadzu equipped with a direct injector. The proposed compound and its fragmentation products can be observed in the measured MS spectrum as indicated by the calculated mass

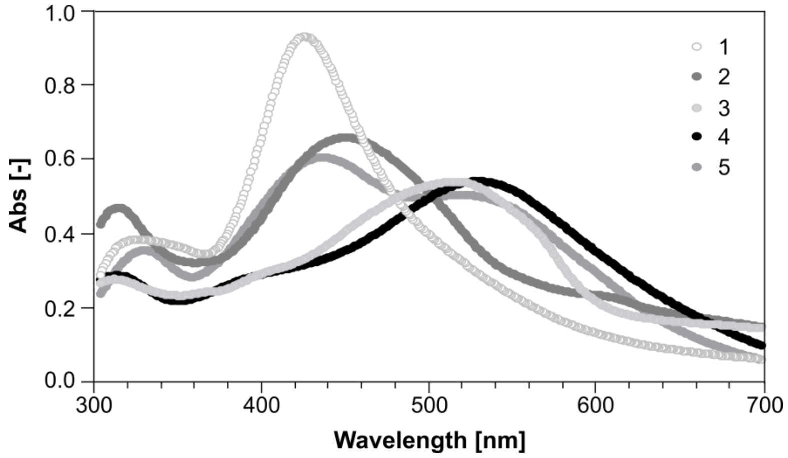

Fig. S3 Absorption scan of different HQs in the presence of NpCN and NaOH. 1 Hydroquinone; 2 Methylhydroquinone; 3 TMHQ; 4 Fluorohydroquinone; 5 Bromohydroquinone

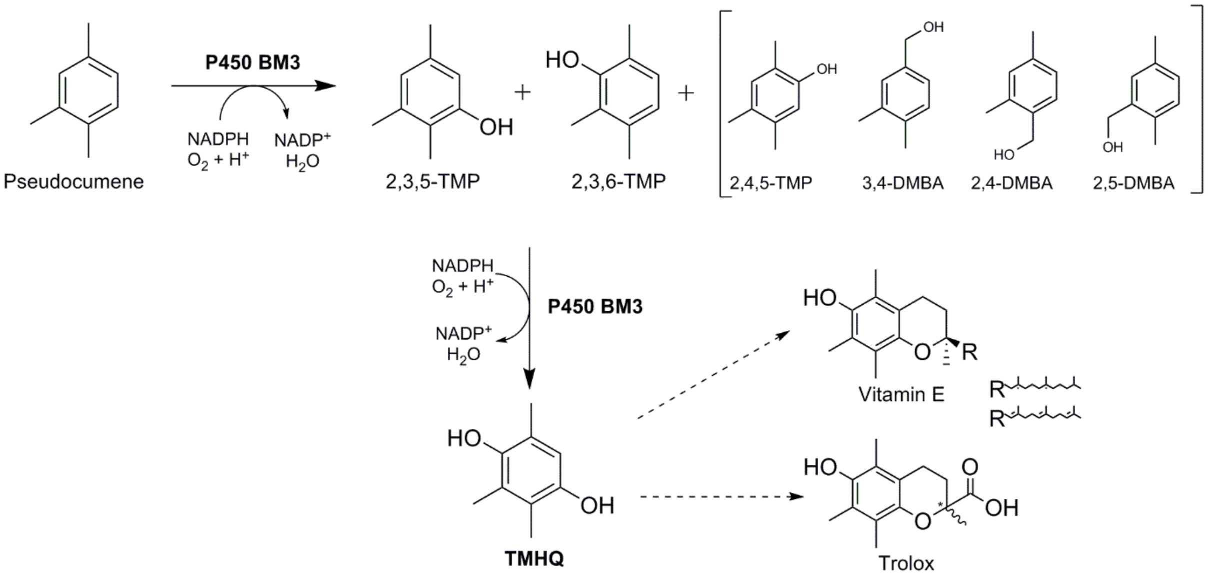

Fig. S4 Hydroxylation of pseudocumene catalyzed by P450 BM3. The intermediates 2,3,5-TMP and 2,3,6-TMP can be further hydroxylated to trimethylhydroquinone (TMHQ) in a one-pot mode. Additional side products formed during conversion of pseudocumene are represented in brackets. TMP, trimethylphenol; DMBA, dimethylbenzylalcohol (Dennig et al. 2017)

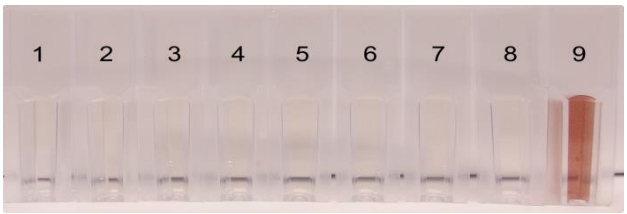

Fig. S5 Different products in the presence of NpCN and NaOH. The products obtained in P450 BM3 catalyzed pseudocumene hydroxylation were investigated separately for their ability to interact with NpCN. 1, phosphate buffer; 2, pseudocumene; 3, 2,3,5-TMP; 4, 2,3,6-TMP; 5, 2,4,5-TMP; 6, 2,4-DMBA; 7, 2,5-DMBA; 8, 3,4-DMBA; 9, TMHQ. NpCN as reactant led to color formation in presence of TMHQ but not with phenols or dimethylbenzylalcohols

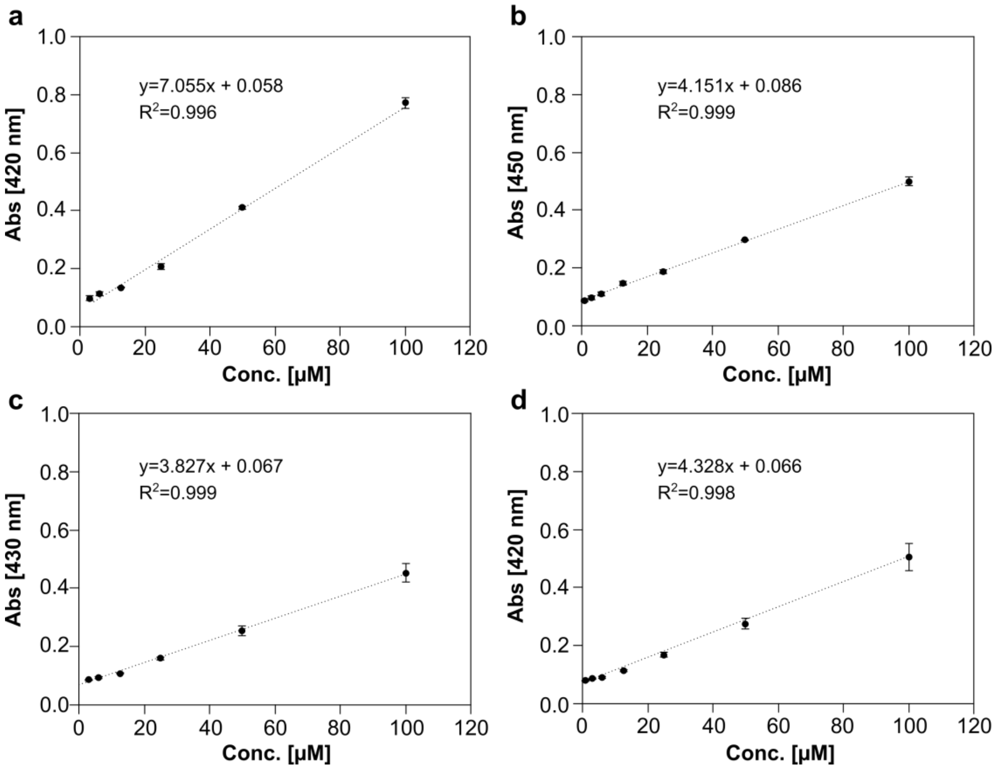

Fig. S6 Linear detection range of different HQs in the presence of NpCN and NaOH in a 96-well MTP format. a, Hydroquinone; b, Methylhydroquinone; c, Bromohydroquinone; d, Chlorohydroquinone

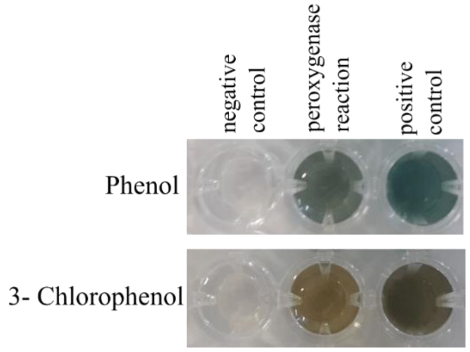

Fig. S7 HQs produced in a peroxygenase catalyzed reaction were detected with the NpCN assay. Reactions contained 20 µL lysate comprising the UPO variant PaDa-I (Molina-Espeja et al. 2014), 5 mM of potential substrate (phenol or 3-chlorophenol), 5 mM H_2O_2 and potassium phosphate buffer (50 mM, pH 7.5, total volume 300 µL). After 20 min incubation, 20 µL (0.04 %) NpCN and 20 µL (2 %) NaOH were added. The negative control did not contain UPO and showed no color formation. In the presence of UPO a color formation was obtained for both substrates. Thus, UPO variant PaDa-I was able to catalyze the aromatic hydroxylation of phenol and 3-chlorophenol. The positive control contained 0.2 mM of the respective HQ

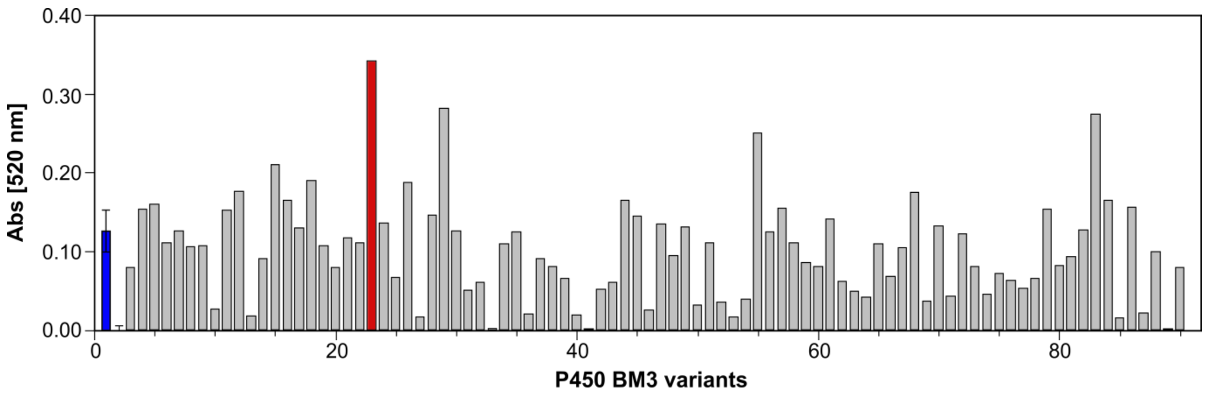

Fig. S8 Screening of the SSM library on position 330 with the NpCN assay. P450 BM3 variant AW1 was used as a starting variant and pseudocumene as substrate. P450 BM3 variants were selected due their ability to produce TMHQ. The blue bar represents the starting variant P450 BM3 AW1. The later characterized P450 BM3 variant AW2 is highlighted in red

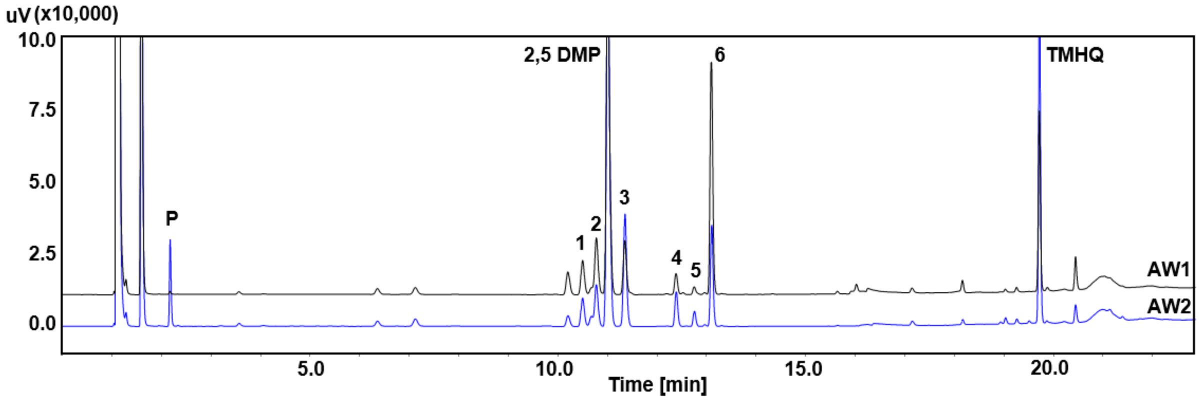

Fig. S9 Product analysis after conversion of pseudocumene with P450 BM3 variant AW1 (black lane) and AW2 (blue lane). The data was obtained from 24 h conversion reactions employing GDH for efficient NADPH cofactor regeneration. 1, 2,5-DMBA; 2, 2,4-DMBA; 3, 2,3,6-TMP; 4, 2,3,5-TMP; 5, 3,4-DMBA; 6, 2,4,5-TMP. An improved TMHQ formation is visible with P450 BM3 AW2 compared to AW1

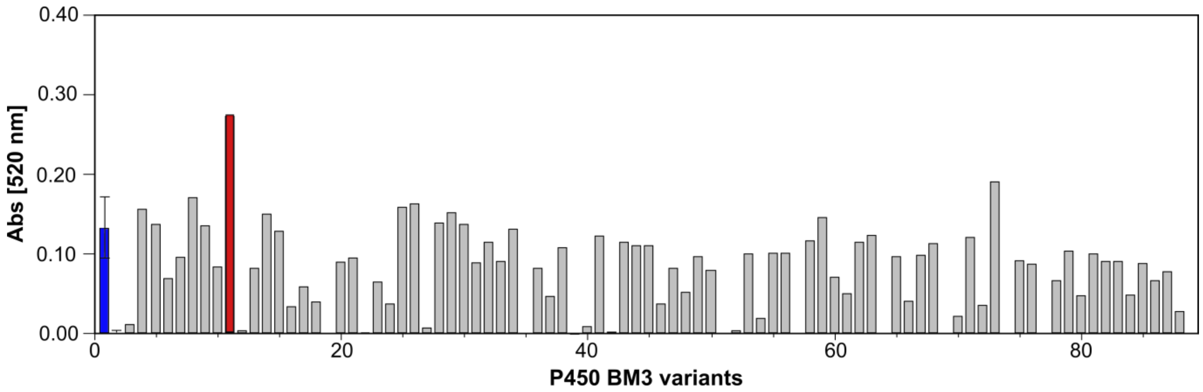

Fig. S10 Screening of the SSM library on position 330 with the NpCN assay using starting variant P450 BM3 M2. P450 BM3 variant M2 (Dennig et al. 2012) was used as a starting variant and pseudocumene as substrate. P450 BM3 variants were selected due their ability to form TMHQ. The blue bar represents the starting variant P450 BM3 variant M2. The later characterized P450 BM3 variant AW3 is highlighted in red

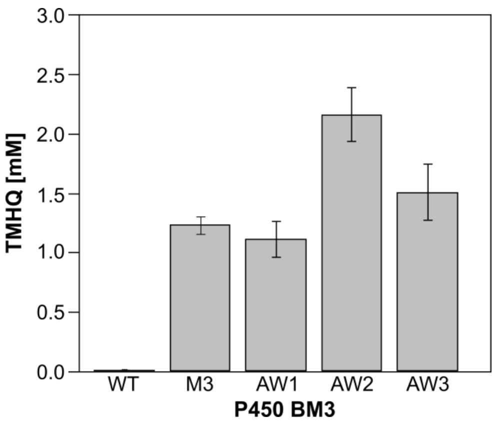

Fig. S11 TMHQ formation of different P450 BM3 variants. TMHQ was detected by GC-FID after 24 h pseudocumene conversion catalyzed by P450 BM3 WT and the variants M3 (Dennig et al. 2017), AW1, AW2 and AW3

Table S1 TMHQ obtained in pseudocumene conversions by P450 BM3 WT and variants

P450 BM3 variants	TMHQ [mM] after 2 h	TMHQ [mM] after 24 h
**AW1**	0.46 ± 0.01	1.11 ± 0.15
**AW2**	0.77 ± 0.06	2.16 ± 0.22
**AW3**	0.52 ± 0.07	1.51 ± 0.24
**M3**	0.49 ± 0.06	1.23 ± 0.09
**WT**	-	0.02 ± 0.01

TMHQ production was determined after 2 and 24 h pseudocumene conversions with GC-FID. The reactions contained 10 mM pseudocumene, 2 % DMSO, cell free lysates (1 µM P450 BM3 WT or variants) and a GDH cofactor regeneration system

Table S2 Selectivity of P450 BM3 variants on the hydroxylation of pseudocumene (in %)

P450 BM3 variants	2,5-DMBA	2,4-DMBA	3,4-DMBA	2,3,6-TMP	2,3,5-TMP	2,4,5-TMP	TMHQ
**AW1**	7	14	2	10	5	37	25
**AW2**	6	13	3	14	5	13	44
**AW3**	7	19	5	14	6	11	37
**M3**	6	18	2	7	4	29	35
**WT**	14	27	5	8	10	34	3

TMP, trimethylphenol; DMBA, dimethylbenzylalcohol. Reaction conditions were: 10 mM substrate, 2 % DMSO, cell free lysates (1 µM P450 BM3 WT or variants), GDH cofactor regeneration system. Conversions were performed at RT and 300 rpm for 24 h

Table S3 Catalytic data for conversion of pseudocumene with purified P450 BM3 enzymes

P450 BM3 variants	NADPH ox. rate [min^{-1}] with pseudocumene	TTN	Coupling eff.
**WT**	63 ± 14	1602	20.8 ± 1 %
**M3**	821 ± 22	4330	40.0 ± 4 %
**AW1**	258 ± 21	5566	31.8 ± 1 %
**AW2**	421 ± 26	7041	37.6 ± 3 %

NADPH oxidation rate ($mol_{cofactor}$ mol_{P450}^{-1} min^{-1}) was determined spectrophotometrically at 340 nm; Coupling efficiency (%) = ratio between product formation [µM] and oxidized cofactor [µM]. NADPH oxidation rate and coupling efficiency were determined using purified P450 BM3. Reaction was supplemented with 1 mM NADPH and activity of P450 BM3 was measured as initial NADPH oxidation rates at 340 nm. The TTN was determined with cell free lysate and calculated based on the product formation after 24 h. Products were quantified using GC-FID and commercial standards

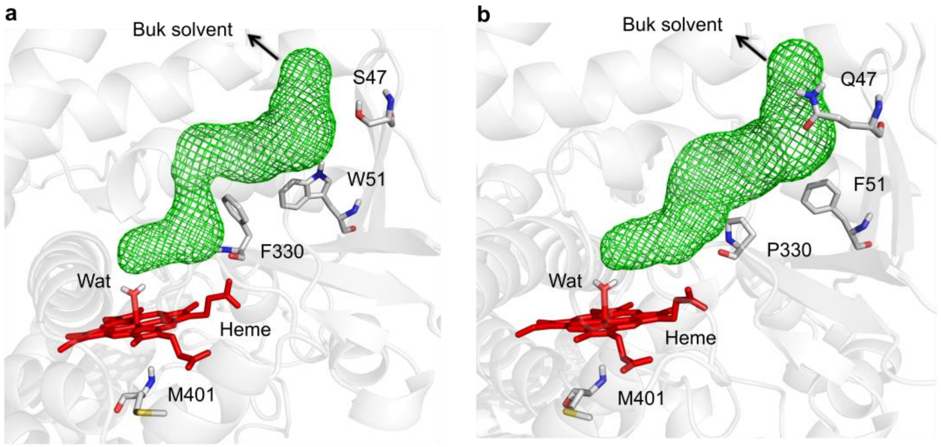

Fig. S12 Molecular docking pose of pseudocumene in the active site of P450 BM3 (a) M3 (R47S, Y51W, I401M, A330F) and (b) AW2 variant (R47Q, Y51F, I401M, A330P). It can be seen from the figure that the F330 in M3 impedes the substrate access channel and P330 in AW2 keeps the substrate access channel open throughout the catalytic mechanism. The substrate access channel is shown as mesh like structure in green, and the residues close to the tunnel are shown as sticks

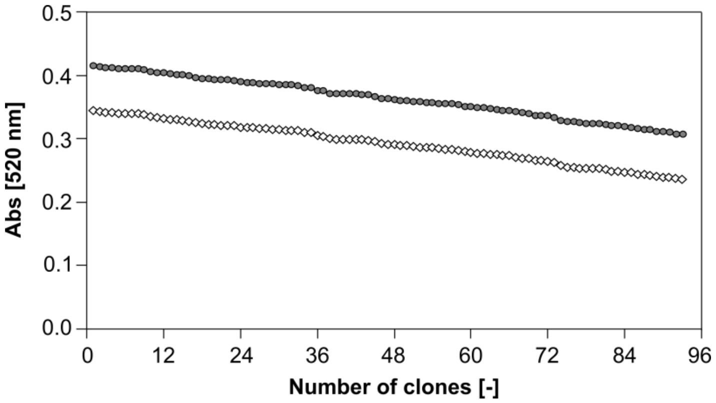

Fig. S13 Standard deviation of the NpCN assay with P450 BM3 AW2. Measured absorption values at 520 nm in descending order of P450 BM3 variant AW2 catalyzed conversion of pseudocumene in a 96-well plate. In dark diamond the apparent standard deviation (10 %) is depicted. The white diamond show the true standard deviation (12 %) after subtraction of the empty vector background

Table S4 Aromatic compounds and their interaction with NpCN

color formation		no color formation	
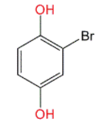	Bromohydroquinone	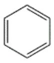	Benzenes
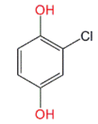	Chlorohydroquinone	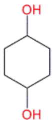	1,4-Cyclohexonediol
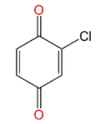	2-Chloro-1,4-benzoquinone	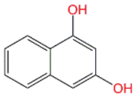	1,3-Dihydroxynaphtalene
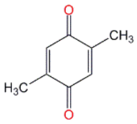	2,5-Dimethyl-1,4-benzoquinone	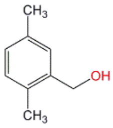	Dimethylbenzylalcohols
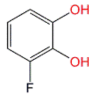	Fluorocatechol	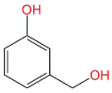	Hydroxymethyl-phenols
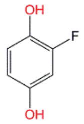	Fluorohydroquinone	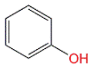	Phenols
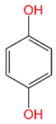	Hydroquinone	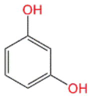	Resorcinol
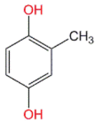	Methylhydroquinone	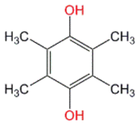	Tetramethylhydroquinone
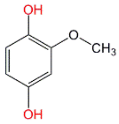	2-Methyloxyhydroquinone		
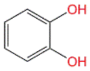	Pyrocatechol		
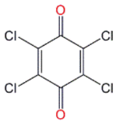	Tetrachloro-1,4-benzoquinone		
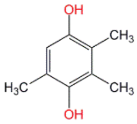	TMHQ		

A color formation of aromatic compounds (1 mM) on the left side was obtained in presence of NpCN under basic conditions. No color formation was visible for the compounds on the right side. Compounds are sorted in alphabetical order

References

Dennig A, Marienhagen J, Ruff AJ, Guddat L, Schwaneberg U (2012) Directed Evolution of P450 BM3 into a *p*-Xylene Hydroxylase. ChemCatChem 4:771 – 773 doi:10.1002/cctc.201200092

Dennig A, Weingartner AM, Kardashliev T, Müller CA, Tassano E, Schürmann M, Ruff AJ, Schwaneberg U (2017) An Enzymatic Route to *α*-Tocopherol Synthons: Aromatic Hydroxylation of Pseudocumene and Mesitylene with P450 BM3. Chem Eur J 23 doi:10.1002/chem.201703647

Fulmer GR, Miller AJM, Sherden NH, Gottlieb HE, Nudelman A, Stoltz BM, Bercaw JE, Goldberg KI (2010) NMR Chemical Shifts of Trace Impurities: Common Laboratory Solvents, Organics, and Gases in Deuterated Solvents Relevant to the Organometallic Chemist. Organometallics 29(9):2176-2179 doi:10.1021/om100106e

Molina-Espeja P, García-Ruiz E, González-Pérez D, Ullrich R, Hofrichter M, Alcalde M (2014) Directed evolution of unspecific peroxygenase from *Agrocybe aegerita*. Appl Environ Microbiol 80(11):3496–3507 doi:10.1128/AEM.00490-14
